# Supplementary material for: Simultaneous Detection of Five Foodborne Pathogens Using a Mini Automatic Nucleic Acid Extractor Combined with Recombinase Polymerase Amplification and Lateral Flow Immunoassay
Source: Microorganisms. 2022 Jul 5;10(7):1352. doi: 10.3390/microorganisms10071352 (PMC9322833; doi:10.3390/microorganisms10071352)
Supplement: Supplementary file 1 [file microorganisms-10-01352-s001.zip › microorganisms-1689555-supplementary.pdf]

Supplementary material:

Figure S1. Optimization of different concentration of each fixed antibody for LFIA

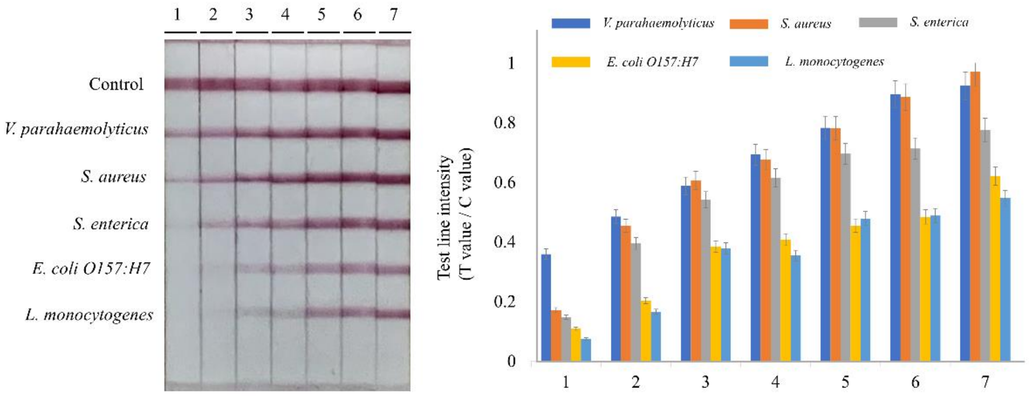

Foodborne pathogens	Fixed antibody	1	2	3	4	5	6	7
V. parahaemolyticus	anti-TET monoclonal antibody	1.5 mg/mL	1.0 mg/mL	0.8 mg/mL	0.7 mg/mL	0.6 mg/mL	0.5 mg/mL	0.5 mg/mL
S. aureus	anti-FAM monoclonal antibody	1.5 mg/mL	0.8 mg/mL	0.6 mg/mL	0.5 mg/mL	0.3 mg/mL	0.25 mg/mL	0.2 mg/mL
S. enterica	anti-TAMRA monoclonal antibody	1.5 mg/mL	1.0 mg/mL	0.8 mg/mL	0.7 mg/mL	0.6 mg/mL	0.55 mg/mL	0.5 mg/mL
E. coli O157:H7	anti-biotin monoclonal antibody	1.5 mg/mL	1.0 mg/mL	0.6 mg/mL	0.5 mg/mL	0.45 mg/mL	0.4 mg/mL	0.4 mg/mL
L. monocytogenes	anti-Cy5 monoclonal antibody	1.5 mg/mL	1.0 mg/mL	0.6 mg/mL	0.5 mg/mL	0.45 mg/mL	0.4 mg/mL	0.45 mg/mL

After commissioning, it showed the final concentration of each fixed antibody in T-lines, including 0.5 mg/mL for anti-TET antibody, 0.2 mg/mL for anti-FAM antibody, 0.5 mg/mL for anti-TAMRA antibody, 0.4 mg/mL for anti-biotin antibody, 0.45 mg/mL for Anti-cy5 antibody. The anti-mouse polyclonal antibody was 2.0 mg/mL in C-line.

Table S1. The recoveries of five foodborne pathogens in spiked food samples by multiple RPA-LFD.

	V. parahaemolyticus				*S. aureus*				*S. enterica*				*E. coli O157:H7*				*L. monocytogenes*			
Samples (n=6 Each)	Inoculation Level (CFU/mL or CFU/g)	RPA-LFIA Detected Concentration (CFU/mL or CFU/g)	Recovery (%)	BMA	Inoculation Level (CFU/mL or CFU/g)	RPA-LFIA Detected Concentration (CFU/mL or CFU/g)	Recovery (%)	BMA	Inoculation Level (CFU/mL or CFU/g)	RPA-LFIA Detected Concentration (CFU/mL or CFU/g)	Recovery (%)	BMA	Inoculation Level (CFU/mL or CFU/g)	RPA-LFIA Detected Concentration (CFU/mL or CFU/g)	Recovery (%)	BMA	Inoculation Level (CFU/mL or CFU/g)	RPA-LFIA Detected Concentration (CFU/mL or CFU/g)	Recovery (%)	BMA
Chicken	3.5×10^{4}	3.45×10^{4}	98.6	+	9.5×10^{4}	9.52×10^{4}	100.2	+	5.2×10^{4}	5.29×10^{4}	101.7	+	8.9×10^{4}	8.71×10^{4}	97.9	+	4.5×10^{4}	4.62×10^{4}	102.7	+
	3.5×10^{3}	3.34×10^{3}	95.4	+	9.5×10^{3}	9.43×10^{3}	99.3	+	5.2×10^{3}	5.41×10^{3}	104.0	+	8.9×10^{3}	8.93×10^{3}	100.3	+	4.5×10^{3}	4.53×10^{3}	100.7	+
	3.5×10^{2}	3.42×10^{2}	97.7	+	9.5×10^{2}	9.43×10^{2}	99.3	+	5.2×10^{2}	5.12×10^{2}	98.5	+	8.9×10^{2}	8.91×10^{2}	100.1	+	4.5×10^{2}	4.38×10^{2}	97.3	+
	3.5×10^{1}	3.40×10^{1}	97.1	-	9.5×10^{1}	8.60×10^{1}	90.5	-	5.2×10^{1}	5.02×10^{1}	96.5	-	8.9×10^{1}	8.62×10^{1}	96.9	-	4.5×10^{1}	4.22×10^{1}	91.6	-
Pork	3.5×10^{4}	3.43×10^{4}	98.0	+	9.5×10^{4}	9.84×10^{4}	103.6	+	5.2×10^{4}	5.32×10^{4}	102.3	+	8.9×10^{4}	8.99×10^{4}	101.0	+	4.5×10^{4}	4.58×10^{4}	101.8	+
	3.5×10^{3}	3.52×10^{3}	100.6	+	9.5×10^{3}	9.75×10^{3}	102.6	+	5.2×10^{3}	4.97×10^{3}	95.6	+	8.9×10^{3}	8.68×10^{3}	97.5	+	4.5×10^{3}	4.34×10^{3}	96.4	+
	3.5×10^{2}	3.22×10^{2}	92.0	+	9.5×10^{2}	9.43×10^{2}	99.3	+	5.2×10^{2}	5.11×10^{2}	98.3	+	8.9×10^{2}	8.93×10^{2}	100.3	+	4.5×10^{2}	4.57×10^{2}	101.6	+
	3.5×10^{1}	3.23×10^{1}	92.3	-	9.5×10^{1}	9.25×10^{1}	97.4	-	5.2×10^{1}	5.18×10^{1}	99.6	-	8.9×10^{1}	8.73×10^{1}	98.1	-	4.5×10^{1}	4.42×10^{1}	98.2	-
Beef	3.5×10^{4}	3.54×10^{4}	101.1	+	9.5×10^{4}	9.93×10^{4}	104.5	+	5.2×10^{4}	5.34×10^{4}	102.7	+	8.9×10^{4}	8.78×10^{4}	98.7	+	4.5×10^{4}	4.53×10^{4}	100.7	+
	3.5×10^{3}	3.42×10^{3}	97.7	+	9.5×10^{3}	9.76×10^{3}	102.7	+	5.2×10^{3}	5.28×10^{3}	101.5	+	8.9×10^{3}	8.88×10^{3}	99.8	+	4.5×10^{3}	4.61×10^{3}	102.4	+
	3.5×10^{2}	3.58×10^{2}	102.3	+	9.5×10^{2}	9.74×10^{2}	102.5	+	5.2×10^{2}	5.37×10^{2}	103.3	+	8.9×10^{2}	8.83×10^{2}	99.2	+	4.5×10^{2}	4.28×10^{2}	95.1	+
	3.5×10^{1}	3.22×10^{1}	92.0	-	9.5×10^{1}	9.43×10^{1}	99.3	-	5.2×10^{1}	5.22×10^{1}	100.4	-	8.9×10^{1}	8.82×10^{1}	99.1	-	4.5×10^{1}	4.38×10^{1}	97.3	-
Milk	3.5×10^{4}	3.46×10^{4}	98.9	+	9.5×10^{4}	9.76×10^{4}	102.7	+	5.2×10^{4}	5.31×10^{4}	102.1	+	8.9×10^{4}	8.97×10^{4}	100.8	+	4.5×10^{4}	4.70×10^{4}	104.4	+
	3.5×10^{3}	3.35×10^{3}	95.7	+	9.5×10^{3}	9.83×10^{3}	103.5	+	5.2×10^{3}	5.23×10^{3}	100.6	+	8.9×10^{3}	8.94×10^{3}	100.5	+	4.5×10^{3}	4.64×10^{3}	103.1	+
	3.5×10^{2}	3.29×10^{2}	94.0	+	9.5×10^{2}	9.84×10^{2}	103.6	+	5.2×10^{2}	5.09×10^{2}	97.9	+	8.9×10^{2}	8.92×10^{2}	100.2	+	4.5×10^{2}	4.55×10^{2}	101.1	+
	3.5×10^{1}	3.46×10^{1}	98.9	-	9.5×10^{1}	9.24×10^{1}	97.3	-	5.2×10^{1}	5.15×10^{1}	99.0	-	8.9×10^{1}	8.87×10^{1}	99.7	-	4.5×10^{1}	4.34×10^{1}	96.4	-
Shrimp	3.5×10^{4}	3.57×10^{4}	102.0	+	9.5×10^{4}	9.64×10^{4}	101.5	+	5.2×10^{4}	5.37×10^{4}	103.3	+	8.9×10^{4}	8.89×10^{4}	99.9	+	4.5×10^{4}	4.52×10^{4}	100.4	+
	3.5×10^{3}	3.56×10^{3}	101.7	+	9.5×10^{3}	9.53×10^{3}	100.3	+	5.2×10^{3}	4.89×10^{3}	94.0	+	8.9×10^{3}	8.84×10^{3}	99.3	+	4.5×10^{3}	4.62×10^{3}	102.7	+
	3.5×10^{2}	3.42×10^{2}	97.7	+	9.5×10^{2}	9.46×10^{2}	99.6	+	5.2×10^{2}	4.99×10^{2}	96.0	+	8.9×10^{2}	8.93×10^{2}	100.3	+	4.5×10^{2}	4.28×10^{2}	95.1	+
	3.5×10^{1}	3.57×10^{1}	102.0	-	9.5×10^{1}	9.67×10^{1}	101.8	-	5.2×10^{1}	5.11×10^{1}	98.3	-	8.9×10^{1}	8.81×10^{1}	99.0	-	4.5×10^{1}	4.26×10^{1}	94.7	-
Fish	3.5×10^{4}	3.25×10^{4}	92.9	+	9.5×10^{4}	9.78×10^{4}	102.9	+	5.2×10^{4}	5.22×10^{4}	100.4	+	8.9×10^{4}	8.98×10^{4}	100.9	+	4.5×10^{4}	4.56×10^{4}	101.3	+
	3.5×10^{3}	3.45×10^{3}	98.6	+	9.5×10^{3}	9.67×10^{3}	101.8	+	5.2×10^{3}	5.28×10^{3}	101.5	+	8.9×10^{3}	8.93×10^{3}	100.3	+	4.5×10^{3}	4.45×10^{3}	98.9	+
	3.5×10^{2}	3.52×10^{2}	100.6	+	9.5×10^{2}	9.45×10^{2}	99.5	+	5.2×10^{2}	5.16×10^{2}	99.2	+	8.9×10^{2}	8.92×10^{2}	100.2	+	4.5×10^{2}	4.35×10^{2}	96.7	+
	3.5×10^{1}	3.53×10^{1}	100.9	-	9.5×10^{1}	9.74×10^{1}	102.5	-	5.2×10^{1}	4.71×10^{1}	90.6	-	8.9×10^{1}	8.89×10^{1}	99.9	-	4.5×10^{1}	4.53×10^{1}	100.7	-

BAM: bacteriological analytical manual. "+": positive result; "-": negative result.

The recoveries in six spiked samples were 92.0-102.3% (for *V. parahaemolyticus*), 90.5-104.5% (for *S. aureus*), 90.6-104.0% (for *S. enterica*), 96.9-101.0% (for *E. coli O157:H7*), and 91.6-104.4% (for *L. monocytogenes*).

Table S2. Comparison of manual extraction and automatic nucleic acid extractor

Sample	Manual extraction		automatic nucleic acid extractor	
	Average nucleic acid content (copies/μL)	Recovery (%)	Average nucleic acid content (copies/μL)	Recovery (%)
Chicken	18739	85.34	20349	92.67
Pork	19348	81.78	22057	93.23
Beef	18064	89.67	18485	91.76
Milk	18995	87.42	20060	92.32
Shrimp	10343	88.69	10736	92.06
Fish	10035	84.77	10797	91.21

The recovery rate was obtained when compared to the standard quantity value.

Table S3. Comparison of the proposed method for foodborne pathogens detection with other methods

Method	Analyte	LOD	Reference
on-chip RPA	*Staphylococcus aureus*<br>*Salmonella enterica*	1×10^{1} CFU/mL	[1]
cPCR-NALF	*Salmonella* Enteritidis<br>*Salmonella* Typhimurium<br>*Escherichia coli O157:H7*	4.5×10^{4} CFU/mL<br>4.5×10^{4} CFU/mL<br>2.3×10^{3} CFU/mL	[2]
mLAMP-LFD	*Salmonella* spp.<br>*Cronobacter* spp.<br>*Staphylococcus aureus*	4.2 CFU/mL<br>2.6 CFU/mL<br>3.4 CFU/mL	[3]
Rti-RPA	*Campylobacteriosis coli*<br>*Campylobacteriosis jejuni*	4×10^{1} CFU/mL	[4]
direct-RPA	*Salmonella enterica*<br>*Escherichia coli O157:H7*<br>*Vibrio parahaemolyticus*	4 cells per 3.2 μL of milk samples	[5]
RPA-LFIA	*Vibrio parahaemolyticus*<br>*Staphylococcus aureus*<br>*Salmonella* Enteritidis<br>*Escherichia coli O157:H7*<br>*Listeria monocytogenes*	2.4×10^{1} CFU/mL<br>7.1×10^{1} CFU/mL<br>4.5×10^{1} CFU/mL<br>5.1×10^{1} CFU/mL<br>2.7×10^{1} CFU/mL	This study

cPCR: convection polymerase chain reaction; NALF: nucleic acid lateral flow; mLAMP-LFD : multiplex loop-mediated isothermal amplification combined with lateral flow dipstick; Rti-RPA: real-time recombinase polymerase amplification

Reference

[1] Kersting, S.; Rausch, V.; Bier, F. F.; von. Nickisch-Rosenegk, M. Multiplex isothermal solid-phase recombinase polymerase amplification for the specific and fast DNA-based detection of three bacterial pathogens. *Mikrochim Acta* **2014**, 181, 1715-1723.

[2] Kim, T.-H.; Hwang, H. J.; Kim, J. H. Ultra-fast on-site molecular detection of foodborne pathogens using a combination of convection polymerase chain reaction and nucleic acid lateral flow immunoassay. *Foodborne Pathog. Dis*. **2019**, 16, 144-151.

[3] Jiang, Y.; Chen, S.; Zhao, Y.; Yang, X.; Fu, S.; McKillip, J. L.; Fox, E. M.; Man, C. Multiplex loop-mediated isothermal amplification-based lateral flow dipstick for simultaneous detection of 3 food-borne pathogens in powdered infant formula. *Int. J. Dairy Sci*. **2020**, *103*, 4002-4012.

[4] Kim, J. Y.; Lee, J.-L. Development of a multiplex real-time recombinase polymerase amplification (RPA) assay for rapid quantitative detection of *Campylobacter coli* and *jejuni* from eggs and chicken products. *Food Control* **2017**, *73*, 1247-1255.

[5] Choi, G.; Jung, J. H.; Park, B. H.; Oh, S. J.; Seo, J. H.; Choi, J. S.; Kim, D. H.; Seo, T. S. A centrifugal direct recombinase polymerase amplification (direct-RPA) microdevice for multiplex and real-time identification of food poisoning bacteria. *Lab Chip* **2016**, 16, 2309-2316.
